# Dengue fatal cases present virus-specific HMGB1 response in peripheral organs

**DOI:** 10.1038/s41598-017-16197-5

**Published:** 2017-11-22

**Authors:** Edson R. A. Oliveira, Tiago F. Póvoa, Gerard J. Nuovo, Diego Allonso, Natália G. Salomão, Carlos A. Basílio-de-Oliveira, Luiz H. M. Geraldo, Celina G. Fonseca, Flávia R. S. Lima, Ronaldo Mohana-Borges, Marciano V. Paes

**Affiliations:** 1 Laboratóio de Modelagem Molecular, Instituto de Química Orgânica, Universidade Federal do Rio de Janeiro, Rio de Janeiro, Brazil; 2 Instituto de Criminalística, Tocantins, Brazil; 3 Ohio State University Comprehensive Cancer Center, Columbus, Ohio, United States of America; 4 Phylogeny Inc, Powell, Ohio, United States of America; 5 Laboratório de Genômica Estrutural, Instituto de Biofísica Carlos Chagas Filho, Universidade Federal do Rio de Janeiro, Rio de Janeiro, Brazil; 6 Laboratório Interdisciplinar de Pesquisas Médicas, Instituto Oswaldo Cruz, Fundação Oswaldo Cruz, Rio de Janeiro, RJ, Brazil; 7 Anatomia Patológica, Hospital Gaffrée Guinle, Universidade Federal do Estado do Rio de Janeiro, Rio de Janeiro, Brazil; 8 Laboratório de Biologia das Células Gliais, Instituto de Ciências Biomédicas, Universidade Federal do Rio de Janeiro, Rio de Janeiro, Brazil

## Abstract

Dengue is an important infectious disease that presents high incidence and yields a relevant number of fatal cases (about 20,000) every year worldwide. Despite its epidemiological relevance, there are many knowledge gaps concerning dengue pathogenesis, especially with regards to the circumstances that drive a mild clinical course to a severe disease. In this work, we investigated the participation of high mobility group box 1 (HMGB1), an important modulator of inflammation, in dengue fatal cases. Histopathological and ultrastructural analyses revealed that liver, lung and heart post-mortem samples were marked by tissue abnormalities, such as necrosis and apoptotic cell death. These observations go in line with an HMGB1-mediated response and raised concerns regarding the participation of this cytokine in promoting/perpetuating inflammation in severe dengue. Further experiments of immunohistochemistry (IHC) showed increased expression of cytoplasmic HMGB1 in dengue-extracted tissues when compared to non-dengue controls. Co-staining of DENV RNA and HMGB1 in the host cell cytoplasm, as found by *in situ* hybridization and IHC, confirmed the virus specific induction of the HMGB1-mediated response in these peripheral tissues. This report brings the first *in-situ* evidence of the participation of HMGB1 in severe dengue and highlights novel considerations in the development of dengue immunopathogenesis.

## Introduction

Dengue is a mild flu-like mosquito-borne illness which presents a small chance (about 5%) of evolving into life-threatening forms^1^. As the causative pathogen, the dengue virus (DENV), is distributed throughout a large extent of the globe, the number of casualties is as high as 20,000 every year^2^. The severe form of dengue is often an abrupt manifestation known as dengue hemorrhagic fever/dengue shock syndrome (DHF/DSS). This complication is marked by thrombocytopenia, hemorrhagic symptoms and increased vascular permeability that sometimes are accompanied by myocardial dysfunction and dehydration^3^. Despite the major epidemiological importance of dengue, little is known about its pathogenesis especially regarding the mechanisms related to the progress from mild to severe disease. Conventional clinical manifestations of dengue (fever, myalgia and headaches) occur during high viremia, however, DHF/DSS takes place after the abatement of fever and virus elimination from the circulation. These observations led to the idea that severe dengue may be developed under an immunopathological basis^4,5^. In light of this assumption, the investigation of immune mechanisms in response to dengue has become a highly attractive research topic, since it may hold the explanation about how severe dengue is established in the host. A number of research groups have already proposed different hypotheses to address the influence of humoral^6–10^ and T cell responses^11–17^ towards the clinical outcome of dengue. However, the comprehension of mechanisms regarding the innate immunity, such as the role of damage-associated molecular patterns (DAMPs) in dengue, is still in its infancy.

DAMPs, also known as alarmins, are host biomolecules that can signal to initiate and perpetuate an inflammatory response^18^. One of these molecules that has drawn attention in virus research is the high motility group box 1 (HMGB1) due to its association to disease severity in dengue^19^ and in other viral infections^20–22^. HMGB1, first described as a DNA binding protein, is now recognized as a key modulator of the innate immunity which also partially regulates the adaptive immunity^23^. HMGB1 typically interacts with DNA structures and while in the nucleus it is involved in multiple functionalities, such as transcription, replication, recombination, DNA repair and genomic stability^24,25^. However, upon cell death (necrosis or apoptosis) HMGB1 can be passively released to the extracellular environment where it acts as a DAMP to activate host defenses^26–29^. On the other hand, immune cells such as macrophages or monocytes when stimulated by pathogen-associated molecular patterns (PAMPs), can actively secrete HMGB1 to the extracellular milieu^30^. Outside the cell, HMGB1 stimulates macrophages and endothelial cells to produce cytokines and chemokines, creating a positive feedback loop of inflammatory responses that may eventually lead to a deleterious scenario^31,32^.

The participation of HMGB1 in dengue as an immune modulator has been reported since 2009, when Kamau and colleagues found that this cytokine can regulate the secretion of TNF-α, IL-6, IL-8 and IFN-α from DENV-infected dendritic cells^33^. HMGB1 has also been correlated with increased vascular permeability, which is a key biological event related to the progress from mild to severe dengue^19,34^. However, little is known about the participation of HMGB1 when considering human samples, especially in the context of severe dengue. Aiming to better elucidate this topic, we investigated HMGB1 expression in peripheral organs of four patients who died from severe dengue. As characterized by histopathology and electron microscopy, liver, lung and heart samples were marked by tissue injuries such as necrosis and apoptotic cell death. Such observations indicated that these samples would be targeted by an ongoing HMGB1-mediated response. Subsequently, as determined by immunohistochemistry (IHC), all these analyzed sites (liver, lung and heart) from dengue fatal cases showed increased expression of cytoplasmic HMGB1, when compared to non-infected controls. *In situ* hybridization together with IHC analyses also revealed co-expression of DENV RNA and HMGB1 within the cytoplasmic region, which suggested a specific participation of the pathogen to promote HMGB1-induced inflammatory processes. This work showed for the first time the *in-situ* characterization and the participation of HMGB1 in severe dengue. Our data highlights the relevance of this cytokine in dengue and brings new considerations concerning the immunopathogenesis of the disease.

## Results

### Post-mortem samples from severe dengue cases show propitious environment for a DAMP-mediated response

In this first segment of the study, we evaluated the microscopic and submicroscopic properties of samples collected from severe dengue cases. The main purpose here was to identify whether these samples presented an environment which is consistent with a DAMP-mediated response. Based on the fact that the liver is one of the main organs affected by dengue^35–38^ and that lungs and heart are also important sites involved with the severe disease^13,37,39–41^, samples from all these three organs were considered.

#### Histopathology

**Figure 1:**
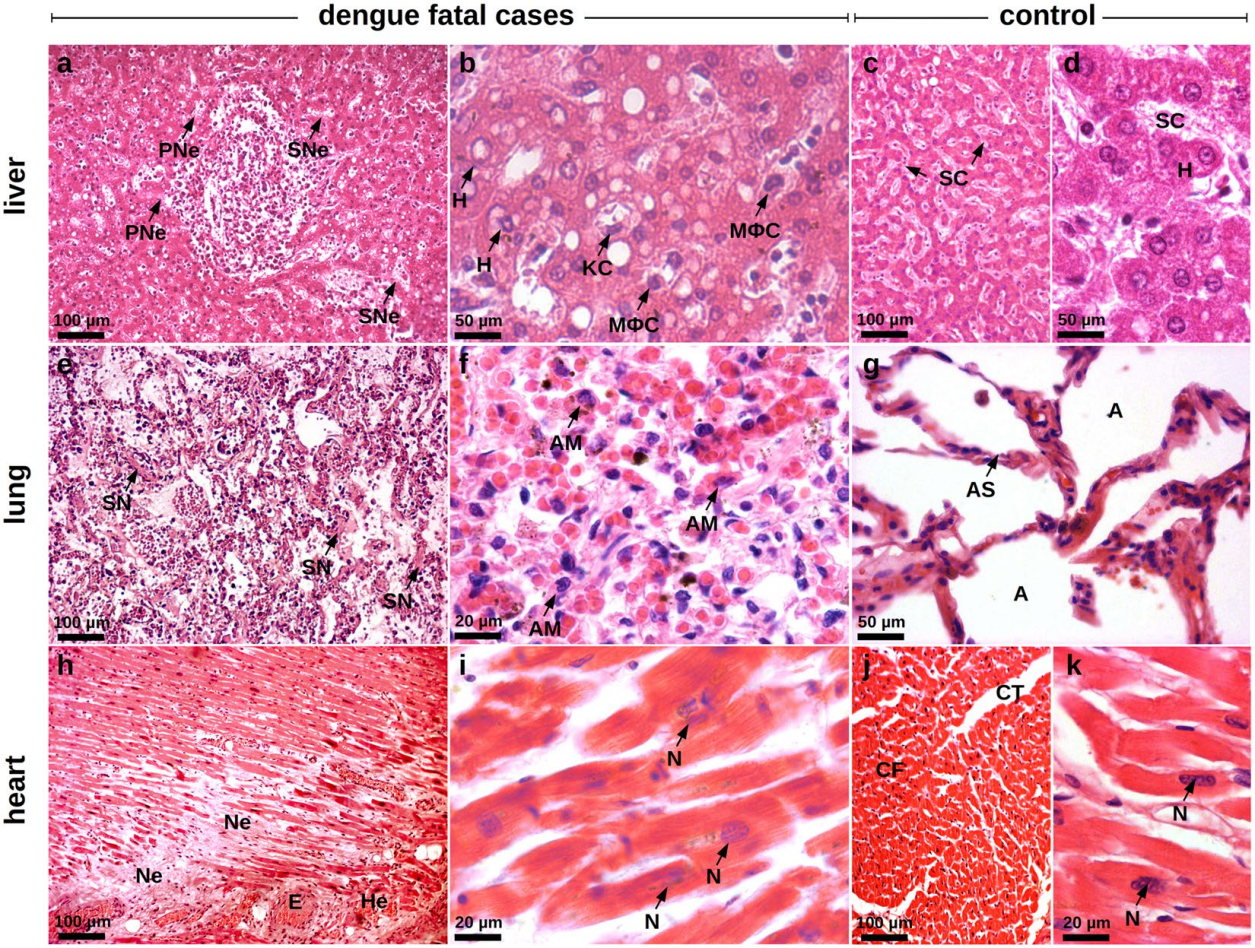
Histological injuries in the peripheral organs of dengue fatal cases (cases 1 and 2). Sections were stained with HE. (**a)** Hepatic parenchyma showing diffuse steatosis. The hepatocytes present swollen and irregular aspects that are classical cellular alterations of necrotic cells in periportal (PNe) and sinusoidal (SNe) areas. (**b)** Sinusoidal region showing hepatocytes (H), Kupffer cells (KC) and circulating macrophages (M*ϕ*C) with chromatin condensation and cytoplasm with bubble-like protuberances. (**c)** and (**d)** Liver sections of a non-dengue case presenting normal hepatic parenchyma (c) and hepatocytes (H) and sinusoidal capillaries (SC) presenting regular aspects of nuclei and cytoplasm (d). (**e)** Pulmonary parenchyma with relevant hemorrhagic areas, hypercellularity, septal thickening and septal necrosis (SN). (**f)** Region of septal thickening presenting numerous alveolar macrophages (AM) with loss of membrane integrity and altered heterochromatin. (**g)** Non-dengue Lung section showing normal alveolar septum (AS) structure and alveolar septal cells with regular characteristics (A - alveoli). (**h)** Extensive areas of necrosis (Ne), edema (E) and hemorrhage (He) in the cardiac parenchyma. (**i)** Cardiomyocytes presenting apoptotic nuclei (N) with heterochromatin damages, loss of membrane integrity and striations. (**j)** and (**k)** Heart sections of a non-dengue case showing (j) normal aspects of cardiac fibers (CF), conjunctive tissue (CT) and (k) cardiomyocytes with regular chromatin distribution (N - nucleus).

Regarding the dynamics of HMGB1 translocation in cell death, it is known that this protein can readily move outside the cell when membrane integrity is lost during necrosis^42^. A Histopathological study of the tissue samples extracted from dengue fatal cases revealed evident necrotic areas that were often accompanied by other related tissue alterations. Liver samples showed several areas within the periportal and the sinusoidal zones that were marked by necrosis. A number of hepatocytes presented swollen and irregular aspects, which are known as classical alterations of these cells when undergoing a necrotic process (Fig. 1a). Similarly, lung tissues from dengue samples were characterized by integrity loss of alveolar septa indicating necrosis (Fig. 1e). Dengue heart samples also revealed considerable damage, as in the cardiac parenchyma we found an extensive destruction of cardiomyocytes probably in function of a high load of lymphocyte and macrophage infiltrates (Fig. 1h).

Similarly to necrotic cells, apoptotic cells can also release HMGB1 and induce inflammation, however, in a time-dependent manner^27,43,44^. Liver samples collected from dengue fatal cases showed several Kupffer cells and circulating macrophages with cytoplasmic blebs and nucleus presenting condensed chromatin (Fig. 1b). In lung samples from DENV-infected patients, the pulmonary parenchyma was marked by numerous alveolar macrophages with altered cytoplasm and heterochromatin (Fig. 1f). Cardiomyocytes also presented a set of morphological damages such as altered heterochromatin distribution patterns, breaks in the integrity of the plasma membrane and the compromise of striations (Fig. 1i). The morphological alterations described above are in congruence with in-progress apoptotic cell death mechanisms and may have implications for a possible HMGB1-mediated signaling. Liver, lung and heart samples that were obtained from non-dengue patients showed regular tissue characteristics (Fig. 1c,d,g,j and k).

#### Ultrastructural findings

**Figure 2:**
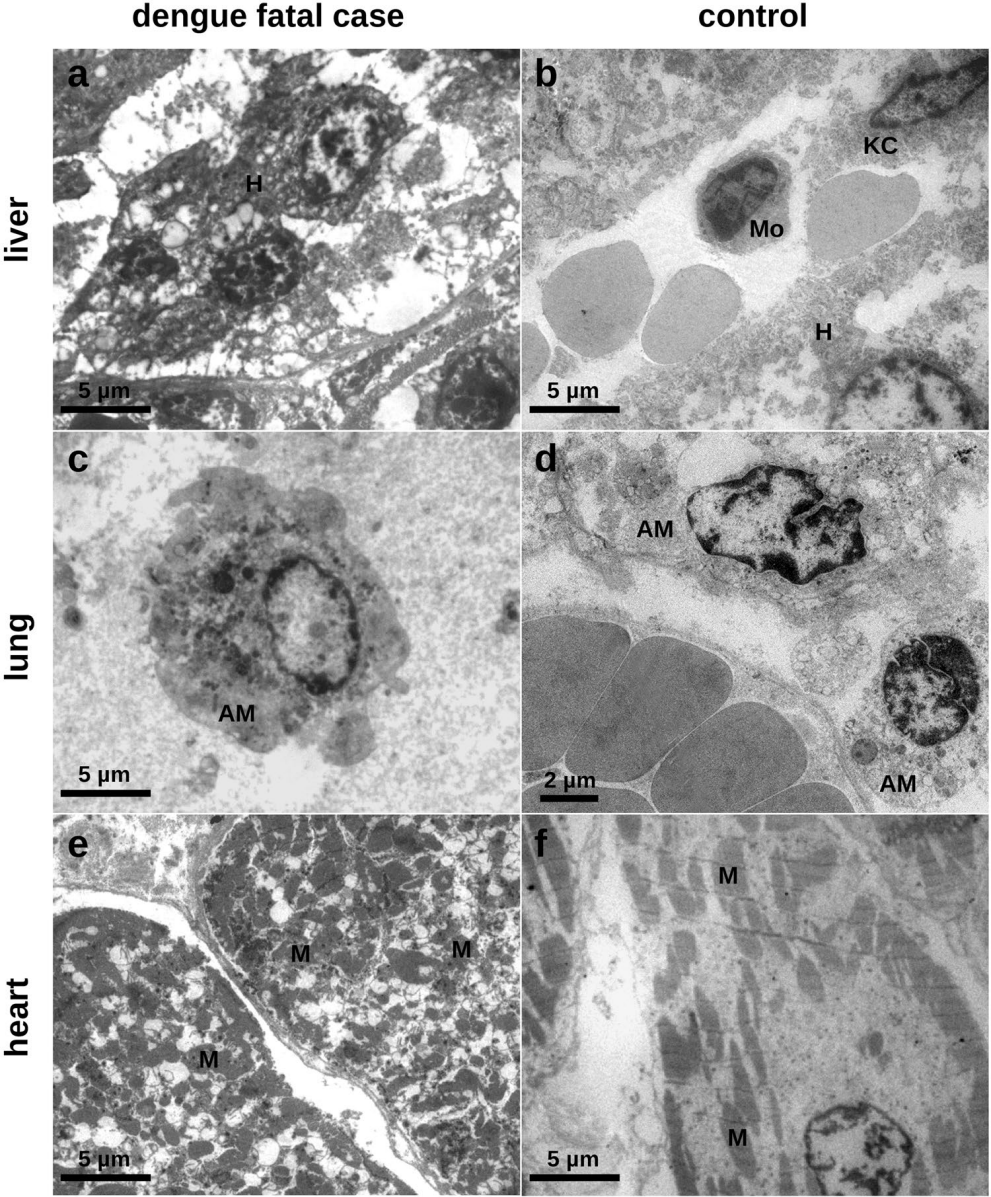
Electron microscopy analysis of peripheral organs collected from a dengue fatal case (case 1). (**a)** Hepatic parenchyma showing hepatocytes (H) with abnormal accumulation of heterochromatin, increased mitochondrias, bleb expansions of the cytoplasm and cell membrane ruptures. (**b)** Non-dengue case showing monocytes (Mo), Kupffer cells (KC) and hepatocytes (H) with regular heterochromatin distribution. (**c)** Alveolar macrophage (AM) exhibiting dense chromatin. The cytoplasmic region is marked by numerous electron-dense granules and loss of mitochondrial integrity. The cell membrane is disrupted at many points also showing bleb-like outward expansions. (**d)** Non-dengue case showing alveolar macrophages (AM) with normal aspects of nucleus and cytoplasm. (**e)** Cardiomyocytes evidencing destruction of the striation patterns and changed spatial distributions of mitochondria (M) in cytoplasmic region. (**f)** Cardiomyocyte from a non-dengue case showing normal distribution of striation and mitochondrias (M).

To provide a more detailed description of the cell alterations found in the tissues from dengue fatal cases, samples were investigated using electron microscopy. Liver sections from dengue cases were mainly marked by hepatocytes presenting damaged cell membrane, increased number of mitochondrias and nucleus with chromatin disorganization (Fig. 2a). DENV lung samples showed alveolar macrophages with nuclei characterized by marginal chromatin condensation. The cell membrane is interrupted at various points and also shows bullet-type outward expansions. Within the cytoplasm we also observed damaged mitochondrial integrity and the presence of several spherical electron-dense granules (Fig. 2c). The electron microscopy analysis also showed details of the destruction of transversal stripes within the cardiomyocytes, which was also accompanied by cell membrane disruption (Fig. 2e). All cells observed in control samples presented normal characteristics (Fig. 2b,d and f).

#### *In situ* cell death assessment

**Figure 3:**
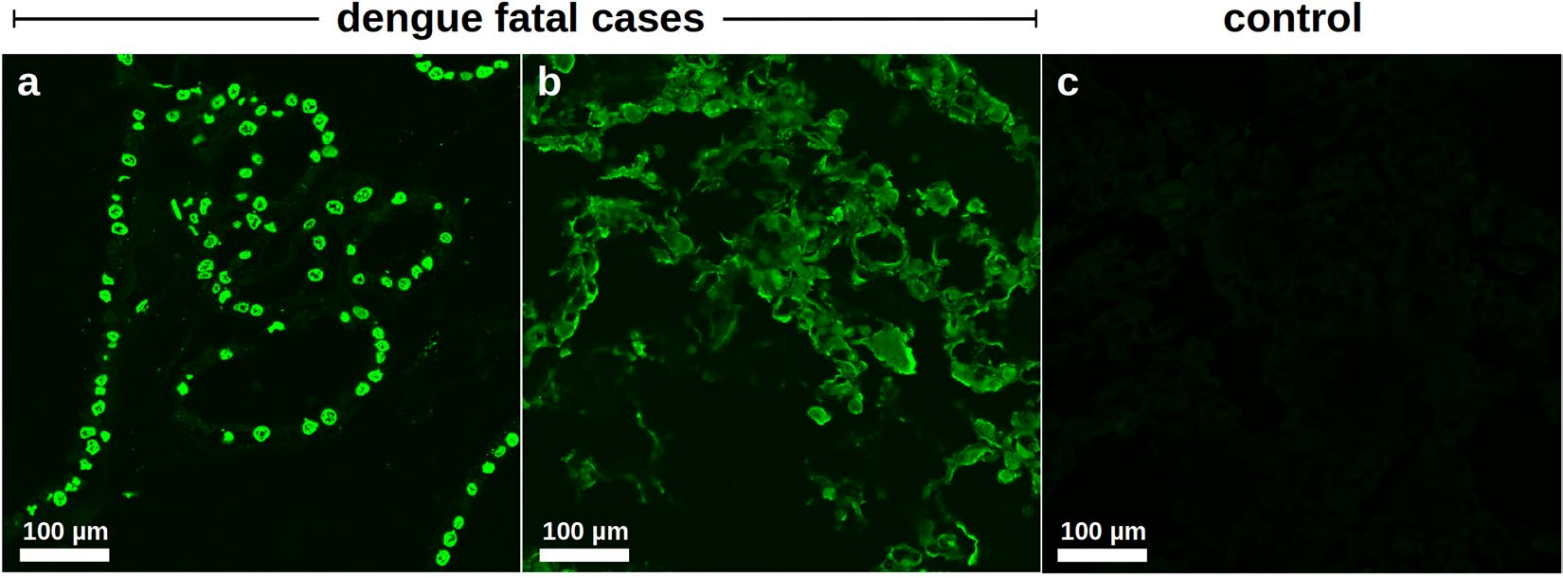
*In-situ* characterization of apoptosis in post-mortem tissues of dengue fatal cases. Apoptosis was assessed by evidencing DNA fragmentation using TUNEL assay. (**a)** and (**b)** Lung sections extracted from dengue cases 1 (a) and 2 (b) evidencing DNA fragmentation (green) in several cells throughout the alveoli. (**c)** Lung section from a non-dengue case exhibiting negative enzymatic reaction for TUNEL assay.

During the late stages of apoptosis, cells evolve to a status in which DNA fragmentation occurs^45^. In addition to the morphological analyses performed above, to provide a more consistent evidence that the organs were importantly affected by apoptosis we assessed DNA fragmentation in dengue post-mortem tissues. Since all the studied dengue cases were affected by respiratory failure and three of them evolved to pulmonary edema, we selected the lung samples for screening with TUNEL assay, an enzymatic-based technique that evidences DNA breaks. As shown in Fig. 3, lung sections extracted from case 1 and case 2 revealed extensive areas of DNA fragmentation. Particularly, in lung sections from case 1 it was clearly seen that alveolar cells were committed by late-stage apoptosis (Fig. 3a). On the other hand, lung sections from case 2 exhibited a diffuse staining which may suggest the occurrence of parenchyma destruction after episodes of apoptotic cell death (Fig. 3b). Liver samples collected from non-dengue cases did not evidence DNA fragmentation by the TUNEL enzymatic reaction (Fig. 3c).

### HMGB1 is over-expressed in peripheral organs of dengue fatal cases

The previous evaluations showed that tissue samples from dengue cases presented alterations that were in accordance with an ongoing HMGB1-mediated activity. Our next step consisted of screening the expression of this cytokine in the three analyzed sites (liver, lung and heart) using immunohistochemistry.

**Figure 4:**
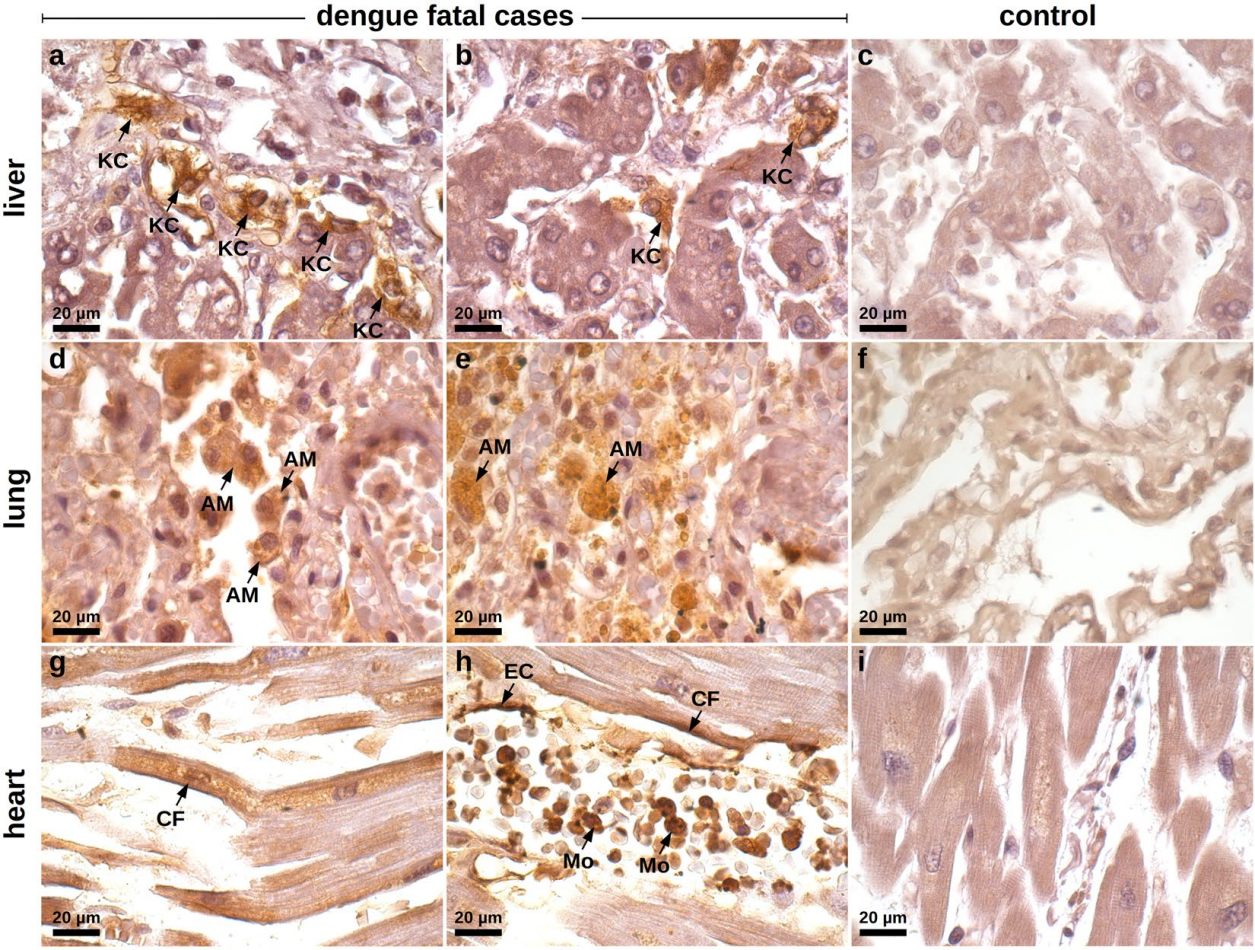
Screening of HMGB1 expression in peripheral organs of dengue fatal cases by immunohistochemistry. (**a)** Hepatic parenchyma of samples collected from dengue case 1 showing hyperplasic and hypertrophic Kupffer cells (KC) with damaged cell membrane and HMGB1 expression within the cytoplasmic region. (**b)** Hepatic parenchyma exhibiting HMGB1 expression in the interior of vesicles of activated Kupffer cells (case 1). (**c**) Non-dengue case showing regular hepatic parenchyma and with basal HMGB1 expression. (**d**) Lung sections showing alveolar septal thickening region containing numerous alveolar macrophages (AM) expressing cytoplasmic HMGB1 (case 2). (**e**) Activated alveolar macrophages expressing HMGB1 inside cytoplasmic vesicles (case 2). (**f**) Liver sections from a non-dengue case presenting regular structure of alveolar septum with a marginal nuclear expression of HMGB1. (**g**) HMGB1 expression along the periphery of necrotic cardiac fibers (CF) (case 1). (**h**) HMGB1 detected in endothelial cells (EC), in the periphery of cardiac fibers (CF) and in the cytoplasm of several monocytes (Mo) present within the blood vessels (case 1). (**i)** Heart sample from non-dengue case showing cardiomyocytes that were negative for HMGB1 staining.

With regards to the liver samples, while controls were clean and did not exhibit a relevant cytoplasmic HMGB1 staining (Fig. 4c), DENV-infected samples showed hyperplasic and hypertrophic Kupffer cells expressing HMGB1 within the cytoplasmic region (Fig. 4a). In general, Kupffer cells presented altered morphology. They were mostly deformed and showed HMGB1 expression in a diffuse pattern, since the cell membrane boundaries were clearly committed by a necrotic process. To a lesser extent, HMGB1 was also detected in the interior of vesicles of activated Kupffer cells, which suggested trafficking of this cytokine to the extracellular space (Fig. 4b). Note that the referred activation was addressed based solely on cell morphology. A similar behavior was observed when analyzing the lung samples. While non-dengue lung samples exhibiting regular structure of the alveolar septum presented only a marginal expression of nuclear HMGB1 (Fig. 4f), in severe dengue samples, which presented alveolar septal thickening, we could observe numerous resident macrophages expressing cytoplasmic HMGB1 (Fig. 4d). The detection of the cytokine was mainly in hypertrophic cells that also presented loss of cellular membrane integrity suggesting necrosis. Monocytes present inside the pulmonary veins as well as some endothelial cells covering these vessels were also positive for HMGB1 within the cytoplasm. In addition to the HMGB1 expression in necrotic cells, activated alveolar macrophages also expressed HMGB1 inside vesicles possibly indicating an active secretion process (Fig. 4e). The aspect of HMGB1 staining in heart samples was more peculiar. While controls were negative and showed a regular structure of cardiomyocytes (Fig. 4i), dengue cases showed HMGB1 expression along the periphery of necrotic cardiac fibers (Fig. 4g). HMGB1 was also detected in endothelial cells and in the cytoplasm of several monocytes present within the blood vessels (Fig. 4h).

**Figure 5:**
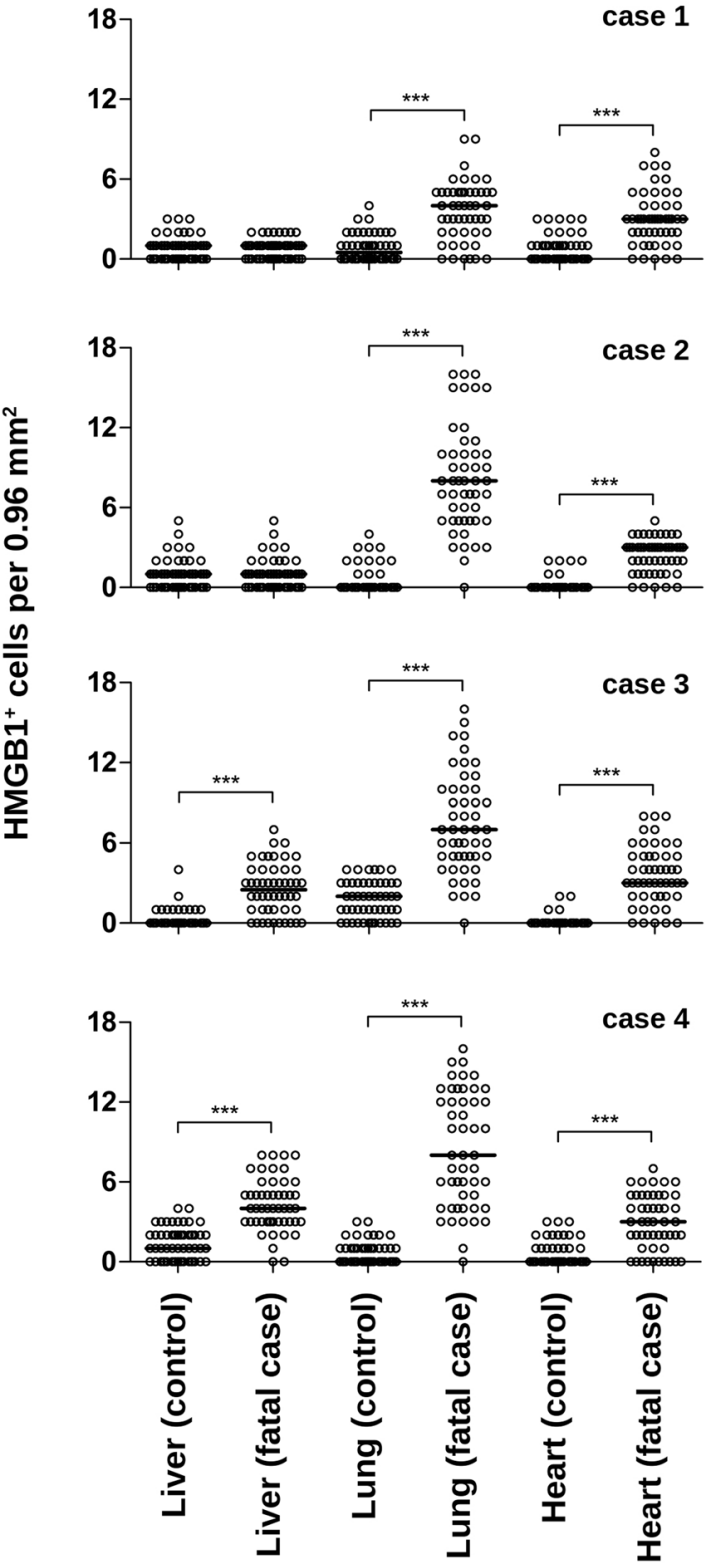
Semi-quantitative analysis of HMGB1^+^ cells within post-mortem tissues of dengue fatal cases. For each studied organ (liver, lung and heart), 50 fields were acquired under a Nikon ECLIPSE E600 microscope coupled with a Cool SNAP-Procf Color camera. Cells with cytoplasmic HMGB1 expression were quantified using organ samples from four dengue fatal cases and four non-dengue control cases. Results are expressed as the median of HMGB1^+^ cells per 0.96 mm^2^. Mann-Whitney non-parametric statistical test was applied to verify the differences between groups (***p < 0.001).

Results from a semi-quantitative analysis revealed that the number of cells expressing cytoplasmic HMGB1 was statistically increased in lung and heart tissues of all four dengue fatal cases, as compared to non-dengue controls. However, concerning the liver, only case 3 and case 4 exhibited relevant increments (Fig. 5). These results clearly showed the translocation of HMGB1 from the nucleus to the cytoplasm and indicated that these studied organs were under the influence of HMGB1 acting as a DAMP.

### DENV-specific induction of HMGB1-mediated activity

Based on the above findings, it remained evident that the samples extracted from dengue fatal cases were under the influence of HMGB1 within the pro-inflammatory context. As immune cells can actively secrete HMGB1 upon PAMP recognition^30^, we were now interested in clarifying whether the dengue infection was triggering the translocation of HMGB1 to the cell cytoplasm. For this, we performed experiments in which HMGB1 protein and DENV genome were co-stained. HMGB1 was detected by immunohistochemical reaction and the viral genome by *in situ* hybridization using a probe that anneals within the viral non-structural protein 3 (NS3) coding region.

**Figure 6:**
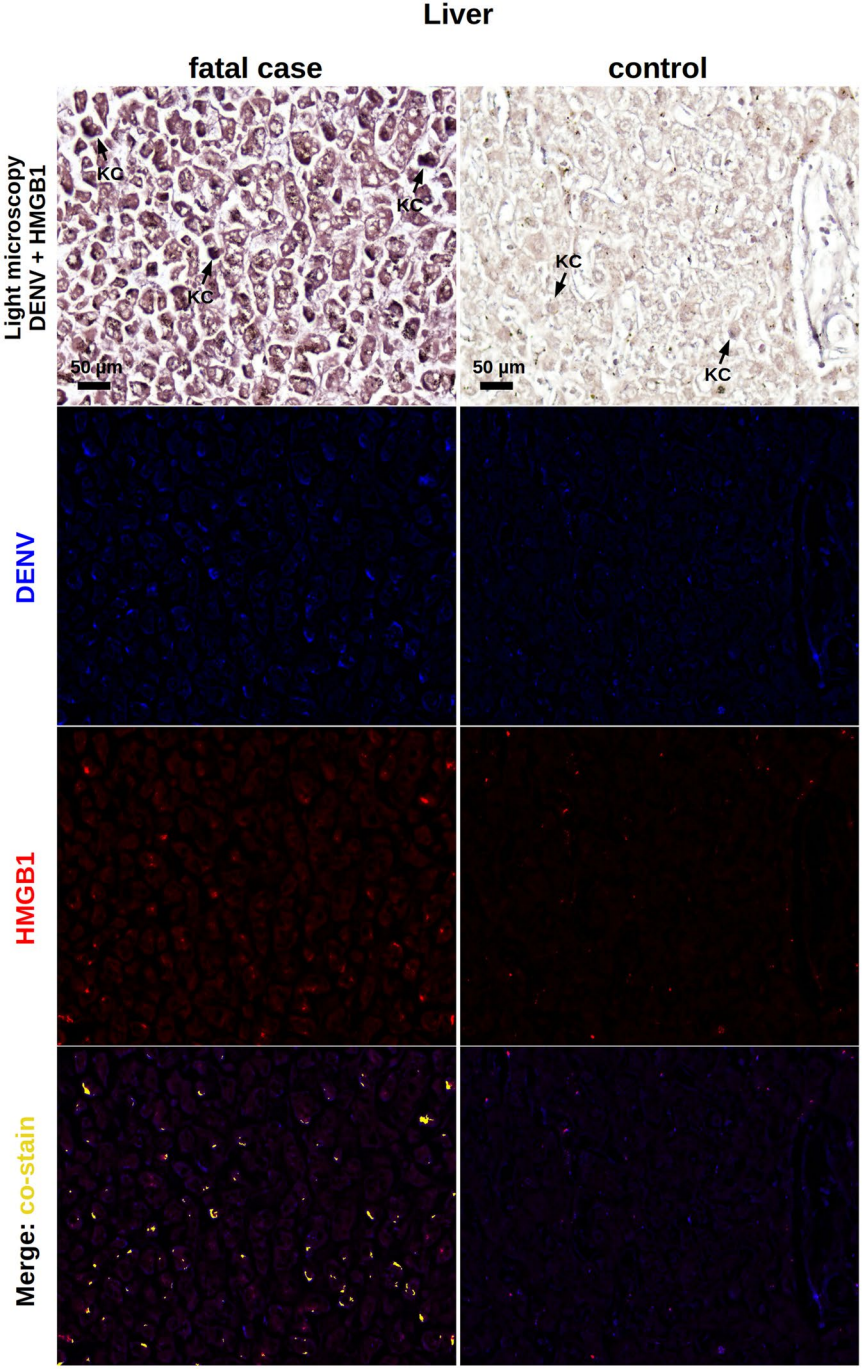
Co-staining of DENV and HMGB1 in the liver. Liver sections from dengue fatal case (case 4) and a non-dengue case were processed for *in situ* hybridization and IHC assays. DENV was detected by a probe that anneals to a conserved sequence within the viral RNA negative strand and HMGB1 was assessed by immunohistochemistry assay. In the dengue case, the figure shows a diffuse area of hepatic steatosis with presence of DENV RNA in Kupffer cells (KC) and in sinusoidal borders of hepatocytes. Expression of HMGB1, when considering the dengue case, occurred within the nucleus as well as the cytoplasmic regions. DENV RNA and HMGB1 signals co-stained within the cytoplasm of hepatocytes and Kupffer cells. In the control case, KCs were negative for DENV and, in general, the HMGB1 expression was marginal and predominantly in nuclear regions.

**Figure 7:**
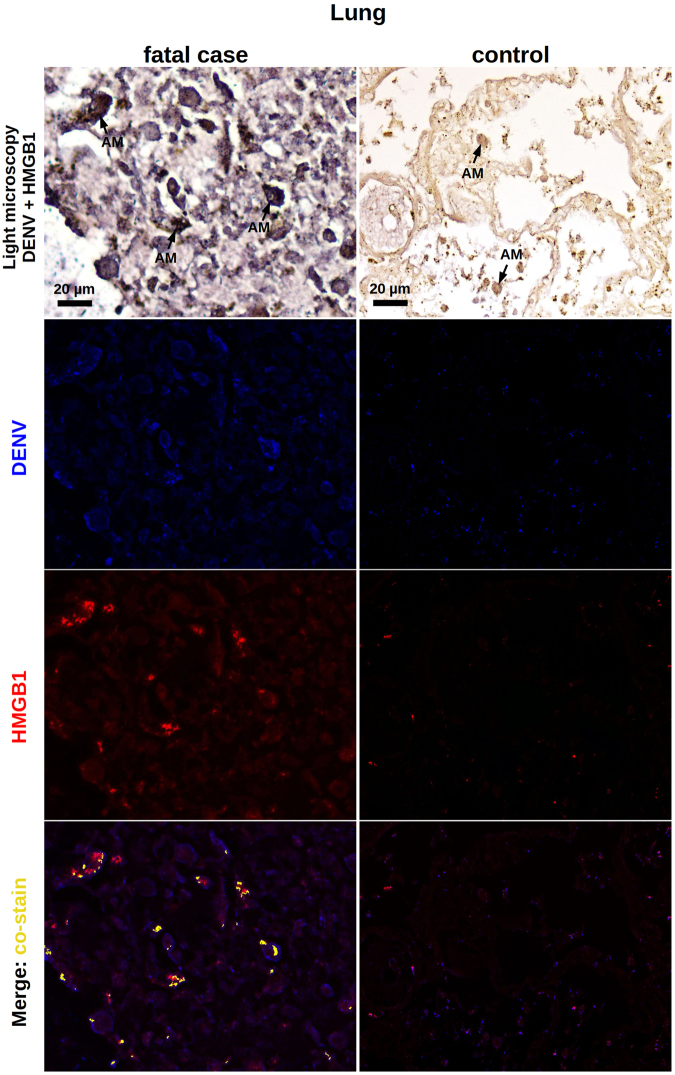
Co-staining of DENV and HMGB1 in the lung. Lung sections from dengue fatal case (case 1) and a non-dengue case were processed for detection of DENV and HMGB1 as explained in Fig. 5. In this dengue case, DENV RNA was found in alveolar macrophages (AM) and in cells from the alveolar septum. HMGB1 expression occurred within the nucleus as well as the cytoplasmic regions. DENV RNA and HMGB1 signals co-stained within the cytoplasm of cells. In the control case, the HMGB1 expression was marginal and predominantly in nuclear regions.

**Figure 8:**
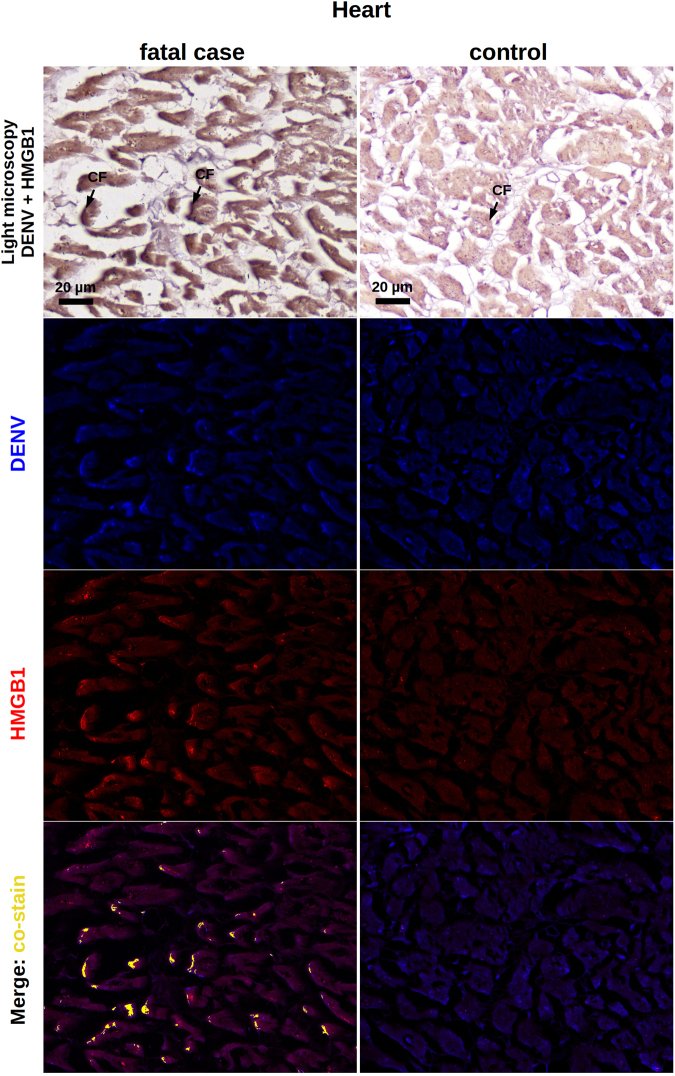
Co-staining of DENV and HMGB1 in the heart. Heart sections from dengue fatal case (case 4) and a non-dengue case were processed for detection of DENV and HMGB1 as explained in Fig. 5. In the dengue case, the figure shows a transversal area of myocardial lesion with integrity loss of cardiac fibers (CF). Viral RNA was found along the periphery of the cardiac fibers and expression of HMGB1 occurred within the nucleus as well as the cytoplasmic regions. DENV RNA and HMGB1 signals co-stained in the periphery of cardiac fibers. In the control case, the HMGB1 expression was marginal and predominantly in nuclear regions.

Dengue liver samples were marked by the presence of viral RNA in Kupffer cells as well as in the sinusoidal borders of hepatocytes. In congruence to what was already seen in this work, the HMGB1 expression occurred either in the cytoplasmic or in the nuclear regions of these cells. We observed several areas of HMGB1 and dengue RNA co-staining, which occurred within the cytoplasmic region of hepatocytes and Kupffer cells (Fig. 6). While analyzing the lung sections extracted from dengue post-mortem cases, particularly, case 1 and 3 showed strong interstitial inflammation. We observed the presence of viral RNA in alveolar macrophages and also in cells of the alveolar septum. As noted before, HMGB1 expression was also detected in the cytoplasm or in the cell nuclei. After merging the fluorescence signals, co-staining of HMGB1 and viral genome was clearly seen throughout the cell cytoplasmic regions (Fig. 7). In transversal heart sections from dengue cases, particularly marked by a relevant myocardial lesion, it was seen the presence of viral RNA in the periphery of cardiac fibers. Co-staining of the viral genome and HMGB1 also occurred in these borders (Fig. 8). All non-dengue controls (liver, lung and heart tissues) were negative for dengue-RNA and showed marginal expression of HMGB1 predominantly within the nuclear regions (Figs 6, 7 and 8).

## Discussion

HMGB1 performs different functions according to its cellular localization. In the nucleus, this cytokine binds genome and is involved in several processes such as transcription, replication, recombination, DNA repair and genomic stability^24,25^. However, when HMGB1 is found in the cell cytoplasm, it suggests its release to the extracellular environment either by passive or active processes. While damaged necrotic or apoptotic cells can passively release HMGB1^26,27^, this cytokine can also be actively secreted from monocytes and macrophages that are stimulated by PAMPs^30^. Once outside the cell, HMGB1 can function as a DAMP and is strictly implicated in the induction or perpetuation of inflammatory processes^30,32^. Inflammation is crucial in the development of dengue disease. When dengue evolves to a severe stage, it is common to see peripheral organs targeted by inflammatory processes that in general are of slow resolution^13,37,46,47^. For this reason, here we investigated the participation of HMGB1 in peripheral organs of dengue fatal cases in an attempt to better characterize dengue pathogenesis.

A first histopathological work with the post-mortem samples was key in guiding our research. We identified that tissue samples (liver, lung and heart) extracted from the dengue cases were marked by injuries such as necrosis and apoptotic cell death (apoptosis was also confirmed by TUNEL assay in lung sections). Since it is well known that necrotic or apoptotic cells can passively release HMGB1^26,27^, this finding represented the first giveaway that the analyzed tissues could be targeted by an ongoing HMGB1-mediated signaling. In sequence, using IHC technique, we screened the post-mortem tissues for the expression of HMGB1. At this point, we found that dengue samples showed increased levels of this cytokine, especially within the cytoplasmic region of cells. More specifically, the HMGB1 staining clearly presented distinct patterns: (i) a diffuse staining pattern, mainly found within necrotic/deformed cells with evidently compromised plasma membranes; and (ii) a condensed staining pattern, observed in the interior of vesicles of activated immune cells. To our understanding, these staining profiles (i and ii) clearly resembled the two contexts of HMGB1 release: the passive release by necrotic or apoptotic cells^26,27^ and the active secretion after PAMP induction^30^, respectively. Although HMGB1 is known to participate in the physiological recovery of damaged tissues^48,49^, the above findings suggested a possible contribution of this cytokine in enhancing inflammation in dengue. Additional experiments of *in situ* hybridization allied to IHC revealed that dengue RNA and HMGB1 co-stain within the cytoplasmic region of immune cells. This finding indicated that, at least in part, the observed over-expression of HMGB1 was triggered under a virus-specific manner.

Together with previous studies from our group, the results exposed here complement the pathological description of the considered dengue cases. This helps us to better understand the deleterious processes that may happen in organs affected by the disease. Two related back-to-back reports showed that these same dengue fatal cases were systemically committed by the infection^37^, and also that several of their peripheral organs were targeted by mononuclear cells^13,37^. Atypically, liver, lung and kidney tissues displayed a cytokine profile with increased levels of pro- (IFN-γ and TNF-α) together with anti- (IL-10 and TGF-*β*) inflammatory elements. Since these sites were largely characterized by damage and distress, one hypothesis to explain this scenario is that the infection led to such a strong local inflammatory process that is was capable of overwhelming the host’s attempts to regulate this signaling. The data obtained in this work corroborates with this idea in the sense that HMGB1, when functioning as a DAMP, is well known to promote and to perpetuate inflammation. In the extracellular environment, HMGB1 can bind to cell surface receptors and provide cytokine-like functions^28,50–53^. As suggested by our co-staining experiments, all four studied dengue fatal cases presented virus-specific HMGB1 signaling in their peripheral organs (liver, lung and heart). Collectively, it means that HMGB1 is probably working in a pro-inflammatory context, inducing and maintaining local inflammation. To a yet unknown extent, HMGB1 may have contributed to the enhancement of organ damage and eventually may have provoked an impact towards the disease progress.

This work also brought up an interesting discussion in examining the relationship between our findings and a biological event considered to be a hallmark in severe dengue: the altered vascular permeability. An increase in vascular permeability occurs prior to plasma leakage and hemorrhage, which are complications that arise from malfunctioning of the vascular endothelium. This means that, during the course of dengue disease, a currently unrecognized mechanism triggered by the infection allows bypassing the strict and complex endothelial machinery that evolved to avoid leakage^54^. The reason why dengue infection induces this hemodynamic compromise has been correlated with pathological immunity. Specific antibody and T cell responses that fail to protect the host against the infection would act misdirectedly and exaggeratedly, resulting in a cascade of cytokine production known as “cytokine storm”^15^. Hypothetically, large amounts of pro-inflammatory and vasoactive cytokines would end up increasing vascular permeability and subsequently leading to hemorrhage^55^. This description is extremely broad and only in recent reports has the understanding of the cellular pathways and the molecular implications towards increased vascular permeability been improved.

Based on recent research, the endothelial hyperpermeability in dengue is apparently resultant from a set of contributing factors that include viral and host elements. Regarding the viral influence, the DENV non-structural 1 (NS1) protein that is present in the circulation of infected patients was demonstrated to disrupt the endothelial glycocalyx layer via induced expression of sialidases, heparanase and cathepsin L^56^. On the other hand, when the host responds to infection with high levels of pro-inflammatory mediators, such as TNF-α, IFN-*γ*-inducible protein 10 (IP-10) and monocyte chemotactic protein 1 (MCP-1), the subject also becomes more exposed to plasma leakage events^57–60^. In this context, HMGB1 can work either by augmenting the host signals or by acting independently to promote leakage. In agreement to this idea, *in*-*vitro* experiments had revealed that HMGB1 can induce the secretion of these same pro-inflammatory mediators (TNF-α, IP-10 and MCP-1), among others^51^. Additionally, HMGB1 secreted from DENV-infected monocytes (K562 cells) can increase the transcellular endothelial permeability of primary endothelial cells (HUVEC cells)^19^. Similarly, isolated HMGB1 was able to induce redistribution of VE cadherin and the formation of intercellular gaps in HUVEC cells^34^. Based on these reports, it would be reasonable to suggest that the HMGB1-mediated signaling combined with DENV NS1 influence (or also with other potential viral antigen) may represent a key consolidation factor for the progression of dengue disease.

During dengue infection, HMGB1 is released to the circulatory system^61,62^ as similarly found in other viral diseases^20,21^. Once the vascular endothelium is exposed to HMGB1 and NS1 they could jointly affect the vascular integrity and increase the chances of triggering plasma leakage and hemorrhage. This makes more sense when we look at the data presented in this manuscript. Considering the four studied dengue fatal cases, three of them (cases 1, 3 and 4) were diagnosed with acute pulmonary edema. The number of HMGB1^+^ cells (presenting cytoplasmic staining) that were counted within the tissues (Fig. 5) indicated that the lung was the main organ targeted by an HMGB1-mediated activity.

HMGB1, working in a pro-inflammatory context and with strong implications in altered vascular permeability, would end up leading to a scenario that is favorable to the progression of dengue disease. We hypothesize that upon a “cytokine storm” event, that usually occurs in secondary infections, the viral influence and a generalized tissue destruction could promote relevant release of HMGB1. This cytokine, in turns, could act cooperatively with other vasoactive cytokines and viral proteins (such as NS1) to enhance the vascular permeability. At this point, events of plasma leakage and hemorrhage would quickly take place in the host. Lung tissues, strongly affected by the excess of fluid would not be able to effectively provide gases exchange leading to respiratory failure. In parallel, the rapid fluid loss to the tissues would result in hypovolemic shock and eventually lead to multiple organ impairment.

Data from this work together with several reports in the literature corroborate with this line of thinking. We observed that peripheral sites (liver, lung and heart) considered as relevant in the context of severe dengue were targeted by HMGB1-mediated signaling. This cytokine, however, has been correlated with bad prognosis in several other situations. Some major examples include: (i) the suggestion of HMGB1 as a serological biomarker of disease severity^21,22,63^; (ii) the implication of HMGB1 in promoting peripheral organ injuries^20,64–67^; (iii) the HMGB1 effect towards augmenting viral replication^68^; and (iv) the influence of HMGB1 in immune modulation which would make the host more permissive to the occurrence of inflammatory processes^69–71^. Additionally, The above considerations emphasize that HMGB1 would be key in dengue pathogenesis and also shed light on novel strategic concepts for therapies against dengue: the blockage of HMGB1 or its downstream signaling targets.

It is important to note that many studies with dengue are based on comparisons between asymptomatic, mild and severe disease. We understand that this group configuration is ideal for drawing conclusions in many research aspects. However, the nature of samples that are generally used in these reports are body fluids (*e.g*. total blood or serum), which makes this group configuration approach feasible. When dealing with tissue samples, in the particular case of dengue, it is extremely difficult to attain this ideal research design. Major reasons for this limitation include ethics in human research: *e.g.* a patient experiencing the debilitating condition of mild or severe dengue would not be able to undergo a liver biopsy due to hemorrhage risk and also because the liver is known to be a major affected organ in dengue^38^. Consequently, the research involving post-mortem tissue samples in dengue usually may present a gap at this point of comparison. Another important limitation that we acknowledge is sampling bias that is particular in studies based only on autopsy samples. These drawbacks make it more difficult to address which observation is specific of severe or mild dengue and, as such, conclusions must be drawn cautiously. However, despite these limitations, we still judge revealing to find out how the picture of such samples in terms of cellular immunity and inflammatory mediators would be. We consider that the existing unanswered questions regarding dengue immunopathogenesis make the post-mortem samples extremely valuable.

Altogether, we envision that HMGB1 could represent an important interplay between elements of the innate immunity and viral-induced adaptive immunity. In a pro-inflammatory context, HMGB1 could act cooperatively with other vasoactive cytokines and viral proteins (such as NS1) to enhance the vascular permeability. Based on the analyses of dengue post-mortem samples, this report highlights the relevance of HMGB1 in dengue and brings novel considerations towards the immunopathogenesis of the disease.

## Methods

### Human cases

All the procedures performed during this work were in accordance to the Ethics Committee of the Oswaldo Cruz Foundation/FIOCRUZ, under the approval number CAEE: 47525115.3.0000.5248 for studies with dengue fatal cases and controls. All the experimental protocols used herein were also approved by the same institutional committee mentioned above. The informed consent was obtained from all subjects.

Human samples used in this study (livers, lungs and hearts) were extracted from dengue fatal cases (viral serotype 3) with origin in Brazil, Rio de Janeiro in 2002. Infections were confirmed by positive serum IgM antibodies. The four negative controls, from both sexes and ranging from 40 to 60 years old, were non-dengue and did not present any other infectious disease. More information about these cases can be found in a previous report of our laboratory^37^. Briefly: **Case 1** - a 63-year-old male patient that developed a sudden onset of headache, myalgia, anorexia and abdominal pain. The case evolved to shock with severe pulmonary congestion followed by death with a clinical diagnosis of dengue hemorrhagic fever. **Case 2** - a 21-year-old female patient who experienced fever, myalgia, headache, nausea, vomiting and diarrhea. The patient evolved to respiratory failure, followed by multiple organ failure and refractory shock. **Case 3** - a 41-year-old female presenting fever, weakness, abdominal pain, leukocytosis, hematocrit of 48% and fluid in the abdominal cavity. The patient was diagnosed with dengue hemorrhagic fever and died from an acute pulmonary edema. **Case 4** - a 61-year-old female that manifested classical dengue symptoms (fever, myalgia, vomiting and diarrhea). The patient evolved to a severe clinical state and died from acute pulmonary edema with sudden cardiac arrest.

### Histopathology

Human samples were fixed in formalin (10%), blocked in paraffin resin, cut in 4 *μ*m, deparaffinized in xylene and rehydrated with alcohol, as described previously^72^. Sections were stained with hematoxylin and eosin (H.E.) and visualized under a Nikon ECLIPSE E600 microscope.

### Electron microscopy

Tissue samples were fixed with 2% glutaraldehyde in sodium cacodylate buffer (0.2 M, ph 7.2), dehydrated in acetone, post-fixed with 1% buffered osmium tetroxide, embedded in EPON and polymerized at 60 °C for 3 days. Semi-thin sections (0.5 mm thick) were obtained using a diamond knife (Diatome, Biel, Switzerland) adapted to a Reichert-Jung Ultracut E microtome (Markham, Ontario, Canada). Sections were stained with methylene blue and blue solution II^73^. Ultrathin sections (60–90 nm thick) were stained with uranyl acetate and lead citrate^74^, and were observed in a Zeiss EM-900 transmission electron microscope.

### Terminal deoxynucleotidyl transferase (TdT)-mediated dUTP nick-end labelling (TUNEL) assay

Apoptosis in formalin-fixed paraffin-embedded (FFPE) tissue samples from dengue fatal cases and non-dengue controls was assessed using Click-iT™ Plus TUNEL Assay for *In Situ* Apoptosis Detection according to manufacturer’s instructions (Thermo Fisher Scientific, CA, USA). Briefly, slices were deparaffinized in Xylene and rehydrated in graded ethanol series (100% twice, 90%, 80%, 70%, 60% and finally 50%) followed by distilled water and phosphate-buffered saline (PBS) wash. Subsequently, TUNEL assay was performed according to manufacturer instructions, slides were counterstained with 4-6-diamino-2-phenylin-dole (DAPI) and coverslipped with fluoromount. Images were acquired using a confocal microscope (Zeiss LSM 510 Meta, Germany) equipped with 40x and 63x NA 1.40 oil-immersion objective.

### Immunohistochemistry (IHC)

For immunohistochemical studies, the paraffin-embedded tissues were cut (sections of 4 μm thick), deparaffinized in xylene and rehydrated with alcohol. Antigen retrieval was performed by heating the tissue in the presence of citrate buffer^75^. Tissues were blocked for endogenous peroxidase with 3% hydrogen peroxidase in methanol and rinsed in Tris-HCl (pH 7.4). To reduce non-specific binding, sections were incubated with blocking solution (Biogen) for 30 min at room temperature. Samples were then incubated over-night at 40 °C with rabbit-produced anti-HMGB1 polyclonal antibody (Abcam, USA). On the next day, sections were incubated with a mouse anti-rabbit IgG, a secondary antibody-HRP conjugate (Spring Bioscience, CA, USA) for 30 min at room temperature. Control sections were stained with a negative control rabbit serum and the secondary antibody. Reactions were revealed with diaminobenzidine (Dako, CA, USA) as a chromogen and the sections were counterstained in Meyer’s hematoxylin (Dako). Finally, samples were examined under a Nikon ECLIPSE E600 microscope.

### Sub-quantitative analysis for HMGB1-expressing cells

Slides were evaluated using a Nikon ECLIPSE E600 microscope with a coupled Cool SNAP-Procf Color camera. For each specific organ, 50 images (fields) were randomly acquired at 400x magnification using the software Image Pro version 4.5. After collecting the frames, cells with cytoplasmic HMGB1 were quantified in each of the 50 fields and the median of positive cell number was determined. All analyzes were accomplished in a blind test without prior knowledge of the studied groups. After quantification, frames exhibited in figures were selected as to be more informative according to specific areas in the analyzed tissues. Data were processed using GraphPad prism software v5.1 (La Jolla, USA) using non-parametric statistical tests. Significant differences between groups (controls and DENV fatal cases) were determined using Mann-Whitney test with ***p<0.001.

### *In situ* hybridization

The assessment of DENV in the sections was performed by *in situ* hybridization using a digoxigenin-tagged probe (5′-TGACCATCATGGACCTCCA-3′) which anneals within the negative strand of the DENV RNA genome, as previously described^37^. Briefly, paraffin-embedded sections of tissues were deparaffinized and digested with pepsin (1.3 mg/ml) for 4 min at room temperature. Tissues were incubated with the probe cocktail at 60 °C for 5 min and then kept overnight at 37 °C for denaturation and hybridization, respectively. Next, samples were washed with 0.2x SSC and 2% bovine serum albumin at 55 °C for 5 min. The probe-target complexes were revealed by the activity of alkaline phosphatase conjugated to anti-digoxigenin.

### Co-staining of DENV RNA and HMGB1

Co-staining of virus and HMGB1 was performed, as previously described^37^, using *in situ* hybridization and immunohistochemistry. Briefly, the DENV probe was first tagged with 5′ digoxigenin and locked nucleic acid (LNA) modified (Exiqon). Resulting complexes were visualized using an antidigoxigenin-alkaline phosphates conjugate and nitro-blue tetrazolium and 5-bromo-4-chloro-39-indolyphosphate as the chromogen. Detection of HMGB1 was then performed by immunohistochemistry (anti-HMGB1, ABCAM ab79823) using Leica Bond Max automated platform (Leica Biosystems) and DAB as the chromogen. No counterstain was done. Data were analyzed by the computer based Nuance system (Caliper Life Sciences, Hopkinton, MA, USA) which separates the different chromogenic signals, converts them to fluorescent-based signals and combine them to determine co-staining.

### Data availability

All data generated or analyzed during this study are included in this published article.
